# MUMAL2: Improving sensitivity in shotgun proteomics using cost sensitive artificial neural networks and a threshold selector algorithm

**DOI:** 10.1186/s12859-016-1341-x

**Published:** 2016-12-15

**Authors:** Fabio Ribeiro Cerqueira, Adilson Mendes Ricardo, Alcione de Paiva Oliveira, Armin Graber, Christian Baumgartner

**Affiliations:** 1 Department of Informatics, Universidade Federal de Viçosa, Viçosa, 36570-900 Brazil; 2 Department of Computing and Construction, Centro Federal de Educação Tecnológica de Minas Gerais, Rua 19 de Novembro, 121, Timóteo, 35180-008 Brazil; 3 Department of Computer Science, University of Sheffield, Western Bank, S10 2TN, Sheffield, UK; 4 Research and Product Development of Genoptix, a Novartis company, 2110 Rutherford Rd, Carlsbad, 92008 USA; 5 Institute of Health Care Engineering with European Notified Body of Medical Devices, Graz University of Technology, Stremayrgasse 16/II, Graz, A-8010 Austria

**Keywords:** Artificial neural network, Cost sensitive classification, Peptide/protein identification, Phosphoproteomics, Shotgun proteomics, Data mining

## Abstract

**Background:**

This work presents a machine learning strategy to increase sensitivity in tandem mass spectrometry (MS/MS) data analysis for peptide/protein identification. MS/MS yields thousands of spectra in a single run which are then interpreted by software. Most of these computer programs use a protein database to match peptide sequences to the observed spectra. The peptide-spectrum matches (PSMs) must also be assessed by computational tools since manual evaluation is not practicable. The target-decoy database strategy is largely used for error estimation in PSM assessment. However, in general, that strategy does not account for sensitivity.

**Results:**

In a previous study, we proposed the method MUMAL that applies an artificial neural network to effectively generate a model to classify PSMs using decoy hits with increased sensitivity. Nevertheless, the present approach shows that the sensitivity can be further improved with the use of a cost matrix associated with the learning algorithm. We also demonstrate that using a threshold selector algorithm for probability adjustment leads to more coherent probability values assigned to the PSMs. Our new approach, termed MUMAL2, provides a two-fold contribution to shotgun proteomics. First, the increase in the number of correctly interpreted spectra in the peptide level augments the chance of identifying more proteins. Second, the more appropriate PSM probability values that are produced by the threshold selector algorithm impact the protein inference stage performed by programs that take probabilities into account, such as ProteinProphet. Our experiments demonstrate that MUMAL2 reached around 15% of improvement in sensitivity compared to the best current method. Furthermore, the area under the ROC curve obtained was 0.93, demonstrating that the probabilities generated by our model are in fact appropriate. Finally, Venn diagrams comparing MUMAL2 with the best current method show that the number of exclusive peptides found by our method was nearly 4-fold higher, which directly impacts the proteome coverage.

**Conclusions:**

The inclusion of a cost matrix and a probability threshold selector algorithm to the learning task further improves the target-decoy database analysis for identifying peptides, which optimally contributes to the challenging task of protein level identification, resulting in a powerful computational tool for shotgun proteomics.

## Background

The goal in proteome studies is to characterize as many proteins as possible in the samples being analyzed, in order to assign to these proteins a role in cellular activities, including cases of severe disease occurrence due to protein malfunction [1, 2]. For this purpose, liquid chromatography coupled with tandem mass spectrometry (LC-MS/MS) is the most commonly used approach [3–5].

An LC-MS/MS run generates thousands of spectra, where each one represents a peptide. The next step is to assign a peptide sequence to each spectrum based on its spectral peak pattern [6]. There are basically two techniques to interpret MS/MS spectra. One is the so-called de novo approach that analyzes the peak patterns without using any external information [7]. The most common technique, however, uses protein sequence databases, which is the case of computational programs such as Sequest and Mascot [8, 9]. These programs perform an in silico digestion of proteins present in the database (DB) and generate virtual spectra from the resulting virtual peptides. Thus, for each observed spectrum, the program finds its best match to a virtual spectrum and the respective peptide sequence is assigned to the given MS spectrum. The programs normally report the ten best matches. Several scores are attributed to a peptide-spectrum match (PSM) to measure its quality [10]. This strategy can be used to identify and quantify peptides/proteins [11]. Nevertheless, a major issue in this procedure is that a single LC-MS/MS run usually leads to thousands of spectra, where fewer than 20% are interpreted correctly [12].

In this work, we are primarily interested in the identification task. In particular, we aim at performing a computational curation of Sequest PSMs, given the enormous volume of spectra that is usually produced and the potentially large number of false positive hits. In this context, it is important to efficiently estimate the false discovery rate (FDR) of the identifications [13].

A common strategy to FDR estimation is the use of a target-decoy database (TDDB) [14]. In this approach, decoy protein sequences are generated to be used along with target protein sequences for the search, which can be performed using a composite target-decoy DB or in two rounds, i.e., one search for each DB (decoy and target). Common methods for generating decoy sequences are to reverse or shuffle the target sequences, keeping the amino acid distribution. The TDDB strategy relies on the premise that the decoy PSMs are good models of the incorrect target PSMs. Hence, for a wrong PSM, the probability of the assigned peptide sequence to pertain to the target DB is assumed to be the same probability of the sequence to pertain to the decoy DB. As a result, a good estimate for the number of wrong spectrum interpretations among target PSMs is simply the number of decoy PSMs [15]. However, even though the TDDB strategy has been used successfully for FDR estimation, it has not been, in general, suitably applied to optimize sensitivity, i.e., more sophisticated combinations of the PSM scores are not fully explored to increase sensitivity [16]. Furthermore, important scores are left out from the FDR estimation process [13].

PeptideProphet is another known approach used to PSM assessment. This method considers mixed statistical distributions of PSM scores to predict correct and incorrect spectrum interpretations [17, 18]. In the case of Sequest PSMs, for example, the Gaussian and gamma distribution parameters for incorrect and correct PSMs, respectively, are estimated by the Expectation-Maximization algorithm [19]. When the dataset presents the assumed distributions, PeptideProphet can provide an accurate probability that a PSM is correct. On the other hand, in certain datasets the scores might present completely different distributions. Particularly in the case of phosphopeptides, the peptide fragmentation process in the MS/MS run is biased towards phosphate groups, which suppresses important ions and leads to odd spectra [20–22].

MUDE and MUMAL are two more recently introduced methods proposed by our group that explore the TDDB strategy without assuming and relying on a data distribution [3, 12]. Both methods describe a more comprehensive use of PSM scores to enhance sensitivity.

MUDE considers in addition to Xcorr and △Cn, normally used in TDDB analyses, four alternative scores: △m, SpRank, and PercIons, provided by Sequest, and RTp-value, provided by the OpenMS proteomics tool [12, 23]. Furthermore, the problem of finding threshold values for the scores that lead to a desired FDR is treated as an optimization problem. Even though it provides a significant increase in sensitivity, the MUDE approach is capable of producing only linear decision boundaries to separate false positives from true positives because the score thresholds are defined individually.

As in MUDE, the MUMAL method to assess PSMs applies a TDDB analysis using a multivariate approach. However, this is accomplished with machine learning techniques, aimed at providing more flexible decision boundaries to further increase sensitivity in the FDR estimation process [3, 6]. MUMAL replaces the optimization procedure in MUDE with an Artificial Neural Network (ANN) algorithm to perform PSM classification. The resulting ROC (receiver operating characteristic) curve is analyzed according to the decoy count idea, i.e., for each point in the curve, the respective discriminant probability threshold *t* is used to count the number of decoy hits with probability (generated by the ANN) equal or greater than *t*. This count is the estimate for the number of target hits with *P* ≥*t* that are incorrect. For the ANN model construction, the training set is the data to be analyzed itself, i.e., all Sequest hits, where the attributes are composed by the six scores mentioned above, and the class labels are: 0 for decoy hits, and 1 for target hits. If, on one hand, all of the decoy hits are obviously wrong, on the other hand, only a minor part of target hits are correct. For this reason, by using classical evaluation methods such as accuracy, precision, and recall, the resulting model is regarded as unsatisfactory because most target hits have characteristics similar to decoy hits. However, the ROC analysis is an optimal tool to find appropriate discriminant probabilities that provide the desired FDR with good sensitivity [24]. Nevertheless, there is room for improving sensitivity even further, particularly concerning the classification procedure, because other techniques could be applied for using decoy hits to characterize the wrong interpretations among target PSMs.

In this vein, we again propose to use ANNs as in MUMAL to keep delineating good decision boundaries. However, two important approaches are included in the PSM assessment procedure. The first one is the use of a cost matrix for making the cost of misclassifying an instance of class 0 (decoy hit) higher than the cost of misclassifying an instance of class 1 (target hit) [25]. It provides a bias toward the correct classification of decoy hits, for which the class labels are definitely correct (decoy hits are obviously wrong). In this way, the incorrect target hits, i.e., the ones with the same characteristics of decoy PSMs, but with different label (class 1), tend to be correctly classified as class 0 by the model. Therefore, decoy hits help to pin down incorrect target hits, providing better decision boundaries, which leads to a higher sensitivity.

The second technique we use for improving the MUMAL approach is to apply a threshold selector algorithm (TSA) [26]. After building the model with an ANN with a cost matrix, and analyzing the ROC curve to see which discriminant probabilities provide suitable FDRs, the discriminant probability that leads to a 1% FDR is selected to be the final threshold value *t* that separates correct from incorrect PSMs. Next, threshold *t* is set to TSA, which replaces the probabilities generated by the ANN approach with probabilities that make more sense in terms of indicating the PSM correctness. For hits with probability = *t*, TSA replaces their probabilities with 0.5, and all other PSM probability values are proportionally normalized, keeping the range [0, 1], so that 0.5 is the point of separation between a set of PSMs with high FDR (those with *P* < 0.5) and a set of PSMs with low FDR (those with *P* ≥ 0.5). Note that the previous version of MUMAL provides a good approach for separating PSMs with low FDR. However, due to the model problem caused by the fact that many class-1 instances have similar characteristics to class-0 instances, the probability value generated by the ANN approach for a target PSM is not appropriate for its individual evaluation. With the probability value adjustment provided by TSA, in turn, individual assessment of PSMs is now possible, which is very important for the protein inference stage, such as the one performed by ProteinProphet [17].

In this work, we performed experiments with 11 datasets, in a comparison with standard methods for PSM assessment, to demonstrate that our method, named MUMAL2, could achieve an average increase of 15% in sensitivity concerning the best current method, for FDRs varying from 0 to 5%. Still, by using Venn diagrams with peptides identified for a 1% FDR, we demonstrated that almost 4-fold more exclusive peptides were found. Furthermore, in an additional experiment using a dataset with known proteins, the ROC area calculated after the adjustment of probabilities by TSA was 0.93, showing coherent probability values. It is worth noting the demonstration of the predictive power of our method for phosphopeptides. In these cases, the score distribution might be very different from non-phosphopeptide PSMs, which complicates the analysis of traditional computational tools such as PeptideProphet.

## Methods

### Datasets

**Fig. 1:**
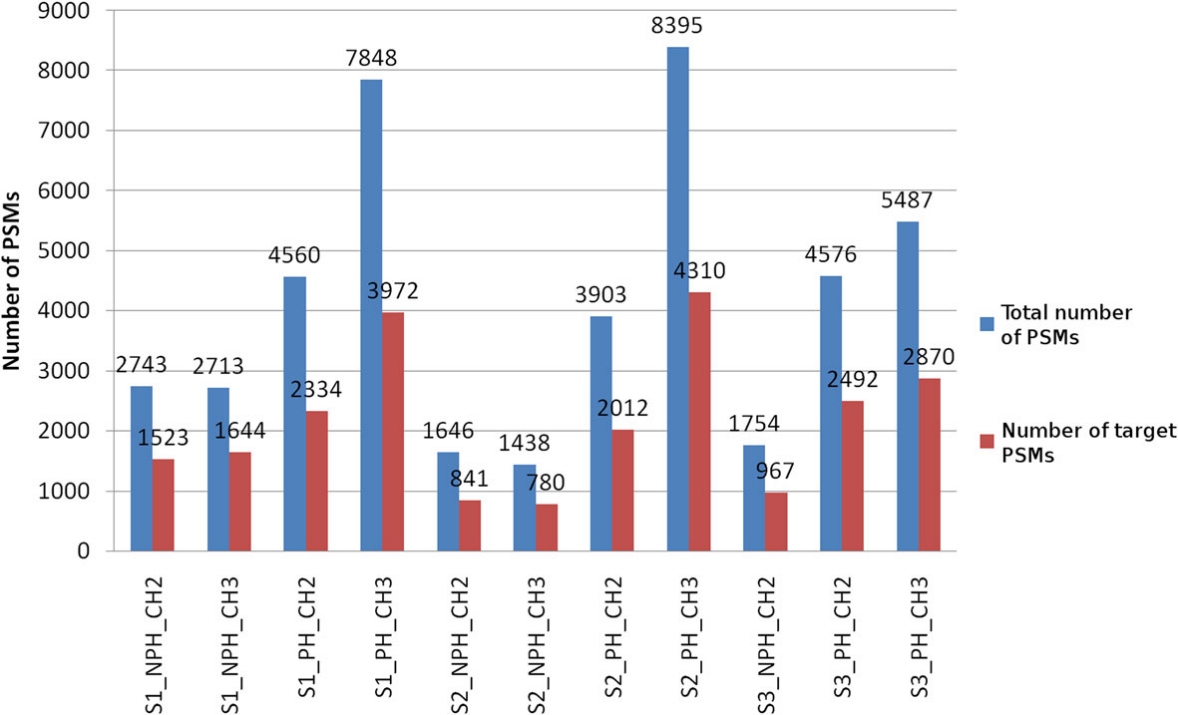
The eleven datasets used in most of our experimental work and evaluation

Eleven datasets used in the validation of MUDE and MUMAL were again utilized in our experiments [3, 12]. Figure 1 illustrates the datasets with their respective amounts of PSMs. Note that in all cases the number of target PSMs is slightly higher than half the total number of PSMs. This is reasonable because it is expected that less than 20% of target hits are correct. Therefore, the total amount of PSMs is composed by this small percentage plus the rest of incorrect PSMs, where, roughly, one half is composed of decoy hits and the other half contains wrong target hits.

These datasets were obtained from three LC-MS/MS runs with three independent phospho-enriched mouse samples. For each resulting set of spectra, Sequest was used for peptide sequence assignment. Each PSM dataset produced as output was split into two parts: The first part contained spectra whose best result was reported as a phosphopeptide, and the second part was made up of spectra whose best hit was attributed to a non-phosphopeptide. These sets were further split based on the precursor charge state, where only +2 and +3 charges were considered. As a consequence, the three Sequest outputs turned into 12 datasets that were labeled S1_PH_CH2, S1_PH_CH3, S1_NPH_CH2, S1_NPH_CH3, S2_PH_CH2, S2_PH_CH3, S2_NPH_CH2, S2_PH_CH3, S3_PH_CH2, S3_PH_CH3, S3_NPH_CH2, and S3_NPH_CH3, where PH and NPH denote phosphodata and non-phosphodata, respectively, while CH2 and CH3 represent +2 and +3 charge states, respectively. The dataset S3_NPH_CH3 was removed from the experiments since it had fewer than ten correct assignments. Finally, for each of these datasets, the Sequest files in the “out” format were converted into a single IdXML file, which is the format used by OpenMS, the computational toolkit we applied to predict retention time (RT) [27]. The details on the protocol and chemicals in sample preparation, MS technology applied, versions of programs and formats, parameters and database used in the Sequest search, etc., can be found in the previous works of Cerqueira et al. [3, 12, 22].

Another dataset we used in our experiments was taken from the work of Pfeifer et al. [23]. They used three samples containing known proteins. In our work, the PSMs of each mixture were also generated by Sequest and were joined in a single IdXML file that we refer to as M123. The proteins present in the mixtures are: *β*-casein (bovine milk), conalbumin (chicken egg white), myelin basic protein (bovine), hemoglobin (human, divided in subunits alpha and beta in the DB), leptin (human), creatine phosphokinase (rabbit muscle), *α*1-acid-glycoprotein (human plasma, appearing in two distinct versions in the DB), albumin (bovine serum), cytochrome C (bovine heart), *β*-lactoglobulin A (bovine), carbonic anhydrase (bovine erythrocytes), catalase (bovine liver), myoglobin (horse heart), lysozyme (chicken egg white), ribonuclease A (bovine pancreas), transferrin (bovine), *β*-lactalbumin (bovine), and thyroglobulin (bovine thyroid). Knowing the proteins we are supposed to identify facilitates the development of experiments to validate the performance of our method to appropriately curate PSMs. The details to produce this dataset can be found in the papers of Pfeifer et al. and Cerqueira et al. [12, 23].

### Target-decoy database strategy

As recommended by Elias et al. [28], we used a composite target-decoy DB for the searches, where decoys were produced by reversing the target sequences. In this way, the peptide sequence of an incorrect PSM has an equal chance of coming from either a target or a decoy sequence. As a result, to estimate the number of wrong target hits, it suffices to count the number of decoy hits, i.e., the FDR estimate for target hits is given by: D _*t*_ /(N _*t*_ - D _*t*_), where D _*t*_ is the number of decoy PSMs found with a score equal or greater than a predetermined threshold *t*, and N _*t*_ is the total number of PSMs (decoys and targets) according to the same threshold *t*.

In order to enhance sensitivity, as proposed previously [3, 12], the TDDB strategy is used here in a multivariate manner, taking into consideration six key PSM scores: △*C*_*n*_, Xcorr, △M, SpRank, percentage of ions found (all of them calculated by Sequest), and RT p-value (calculated by OpenMS). A careful statistical analysis was previously performed to evaluate the impact of each of these scores on PSM curation [12].

In addition to the multivariate approach, machine learning techniques are applied to promote a better separation between correct and incorrect hits, using decoy PSMs as a key part in this procedure, as described in the next sections.

### Cost sensitive artificial neural network

**Fig. 2:**
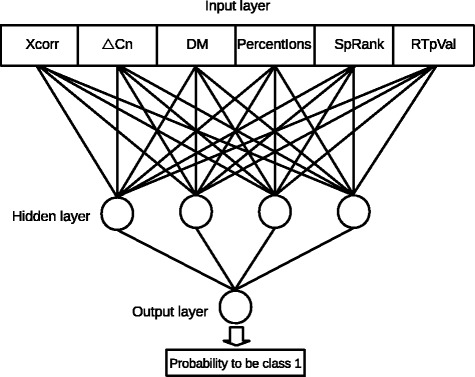
Architecture of the artificial neural network used in MUMAL and in this work. It illustrates the input layer (*six nodes*), the hidden layer (*four nodes by default*), and the output layer (*one node*), where the activation function is the sigmoid to map the ANN’s output into a value in the range [0, 1]

Classical decoy approaches typically use no more than two scores that have their threshold values analyzed individually. Such a procedure leads to linear decision boundaries. In order to construct more appropriated decisions boundaries between correct and incorrect hits, an ANN is used so that the six scores (the ANN’s inputs) mentioned previously are applied in combination to produce a final score in the range [0, 1] (the ANN’s output using a sigmoid function) that can be interpreted as a probability value. Then, using the decoy counting idea, a suitable threshold for this score is pursued to reach a desired FDR. Figure 2 illustrates the ANN architecture.

In the MUMAL work, the authors compared support vector machines with ANNs and showed that the latter approach was capable of delivering higher sensitivity. The authors still observe that labeling decoy PSMs as class 0 and target PSMs as class 1 leads to a difficult classification task because most target PSMs are incorrect, i.e., they are similar to decoy hits. However, the goal is not providing a perfect separation between class-0 and class-1 instances. Once a model (even supposed of low quality using traditional metrics such as accuracy) is created, different discriminant probabilities are tested to obtain one that results in a sought FDR. This threshold exploitation using decoy counting is the key to separate what really matters, i.e., correct from incorrect hits among target PSMs.

In this work, we improve the MUMAL approach to further increase the sensitivity in PSM assessment. The selected strategy is to use the decoy instances in the data-set to pin down wrong target instances, so that the model’s capacity to separate correct from incorrect hits is improved. For this purpose, a cost matrix is introduced to the classification task [25, 26, 29], where, considering target instances as positives, the cost of a false positive (CFP) is set higher than the cost of a false negative (CFN). Therefore, the final model will tend to classify class-0 instances correctly, while class-1 instances will be mostly “misclassified”. The double quotes are to call the attention to the fact that the final goal is to construct a model to separate correct from incorrect PSMs, not separating target from decoys. Hence, when most target instances are classified as class 0 by the model, they are being, actually, correctly relabeled to class 0 because their peptide sequences were incorrectly assigned. Table 1 shows an example of a cost matrix that forces the model to favor decoy instances. The idea is to provoke a model bias toward decoy instances, leading to the relabeling of wrong target instances to class 0, resulting in better decision boundaries to separate correct from incorrect PSMs.

**Table 1:** Cost matrix for a 2-class (class 0 and class 1) classifier

		Predited class	
		0	1
Given class	0	CTN=0	CFP=10
	1	CFN=1	CTP=0

In this case, the cost of a false positive is 10 times higher than the cost of a false negative. CTN = cost of a true negative, CFP = cost of a false positive, CFN = cost of a false negative, and CTP = cost of a true positive

As can be seen in the results of our experiments, this relabeling process could be successfully accomplished. However, after fixing CFN = 1 and trying different values for CFP, we have realized that a certain CFP that leads to a good model for a given dataset is not necessarily the best choice for another dataset. As a result, for each dataset given as input to our pipeline, ten different models are created varying CFP with the integer values in the range [1, 10], and many discriminant probabilities resulting in different FDR values for each case are reported. Next, the model with the highest average number of correct PSMs, for FDRs varying from 1 to 5%, is selected as the final classifier. This cost sensitive classification was implemented in the Java programming language using the Weka API v3.7.8 [26, 30].

### ROC curve

As already mentioned, several discriminant probabilities are explored after the model construction, so that the count of decoys considered as positives serves as an estimate to the number of wrong positive targets. This task is accomplished by analyzing the resulting ROC curve [25]. For each point in the curve, the respective discriminant probability *t* is used to count the number of decoy hits with probability (generated by the ANN) equal or greater than *t*. This count is the estimate for the number of target hits with *P* ≥*t* that are incorrect. As described before, class labels in the datasets are: 0 for decoy hits, and 1 for target hits. It is expected that a minor part of target hits are correct. For this reason, by using classical evaluation methods such as accuracy, precision, recall, and area under the curve (AUC), a very poor classification model is expected. However, we stress the fact that the goal is not separating decoys from targets, but incorrect from correct hits, and the ROC analysis suffices to establish appropriate discriminant probabilities that provide the desired FDR with a better sensitivity when compared to classical target-decoy approaches.

**Fig. 3:**
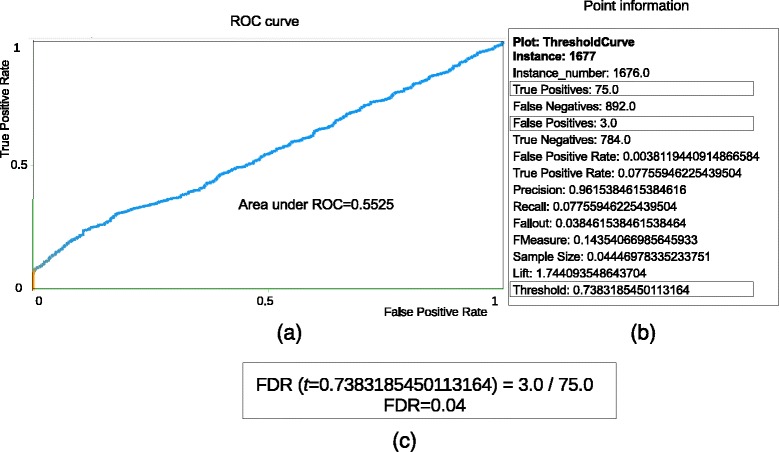
Analysis of a ROC curve obtained from a model built with dataset S2_NPH_CH2. For each point of the curve **a**, the threshold and several statistical measures **b** related to that point are known. False and true positives are, respectively, instances of class 0 (decoys) and class 1 (targets) that were considered positives (*P* ≥ threshold) by the model. In **c**, it is shown how to estimate FDR among target hits for a given discriminant probability (threshold) of the model. It is simply the number of FPs over the number of TPs

Figure 3 illustrates such an analysis. For each point in the curve (Fig. 3 a), the discriminant probability *t* (threshold) and the respective statistics (Fig. 3 b) are known. The FDR estimation (Fig. 3 c) is made by dividing the number of FPs by the number of TPs because FPs and TPs are, respectively, the decoys and targets with *P* ≥*t*.

### Threshold selector

We could see that the ROC analysis gives us the chance of selecting an appropriate probability threshold value leading to a desired FDR. This is enough if the goal is just the selection of a low-FDR set of PSMs. Nonetheless, such a set is normally used in the protein inference stage, where the proteins in the sample being analyzed are ultimately identified. To this end, the probability associated to each PSM is of great importance, mainly for computational tools that use this value as a key measure to infer proteins, e.g., ProteinProphet. On the other hand, the probability values originally assigned by the ANN to the instances in the dataset do not reflect the correctness of PSMs. These values indicate, instead, whether PSMs are decoys or targets because the class labels where defined this way. Considering that most targets (the incorrect ones) are similar to decoys, even the distinction of decoys and targets is not very well characterized in these probabilities. The AUC = 0.55 seen in Fig. 6 clearly shows this fact.

In order to obtain appropriate probability values indicating PSM correctness, we came up with another improvement by using the threshold selector algorithm (TSA) implemented in the Weka API. This algorithm can work in two ways. In the first setting, TSA automatically finds a discriminant probability that optimizes some given measure such as F-measure, accuracy, precision, and recall. In the second setting, TSA is given a fixed threshold value. Then, TSA forces the classifier to predict as positive all instances with probability greater or equal to the given threshold, or as negative, otherwise. Our pipeline uses the latter option along with the probability range correction that TSA provides. In this correction procedure, TSA replaces the probabilities that are equal to the given threshold with 0.5 and expands the other values so that the minimum probability observed maps to 0, while the maximum maps to 1.

The threshold given to TSA is defined as follows. After building a model with a cost-sensitive ANN, with the best cost matrix, and analyzing the ROC curve to perform FDR estimations, the discriminant probability that results in 1% FDR is selected to be the final threshold value *t* that separates correct from incorrect PSMs. We have chosen 1% because this is the best trade-off between sensitivity and precision, as described by Elias et al. and Balgley et al. [31, 32]. Next, the threshold *t* is given to TSA that adjusts the probabilities generated by the ANN, producing new values that are more appropriate to indicate the PSM correctness. For PSMs with P = *t*, TSA replaces their probabilities with 0.5. All other PSM probability values are modified as described above. As a result, we finally obtain a classifier that separates correct from incorrect PSMs (not target from decoys) with the usual mid-point probability value 0.5 as the point of separation between negatives and positives.

### Framework

**Fig. 4:**
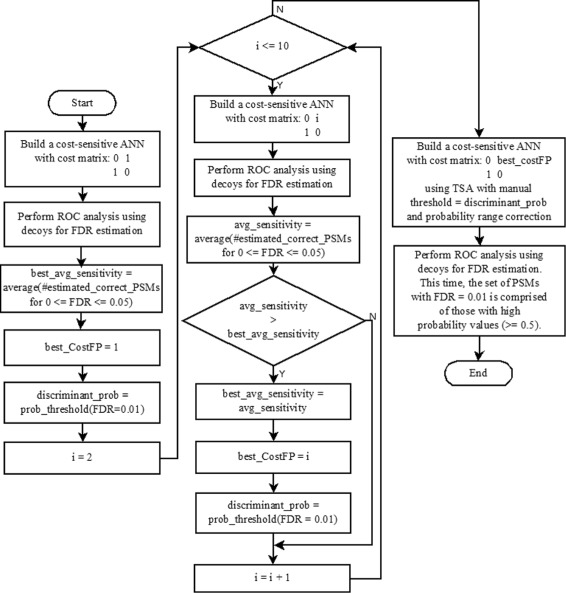
Flowchart to illustrate MUMAL2’s framework. Ten different values for the cost of a false positive are tested in the cost matrix. After selecting the best value in terms of the resultant sensitivity, the final model is built, including the use of TSA with probability range correction. The probability threshold for a 1% FDR identified in the ROC analysis is converted to 0.5 by the TSA. Therefore, the end model uses 0.5 as the discriminant probability, i.e., the set of PSMs with FDR = 0.01 are characterized as the ones with high probabilities (≥ 0.5)

Figure 4 shows a flowchart that summarizes MUMAL2’s framework. Ten cost-sensitive ANNs are built for CFP varying from 1 to 10. Then, the best CFP is selected according to the execution with the highest sensitivity. Furthermore, the probability threshold leading to FDR=1% of the best execution is saved. A final model is thus built with the best CFP and using TSA with the saved threshold. TSA makes a range correction, where PSMs with probability equal to the saved threshold have their probabilities replaced with 0.5. Additionally, the other probability values are expanded, such that the minimum probability is set to 0 and the maximum is set to 1. As a consequence, the PSMs with FDR=1% are assigned high probabilities (≥ 0.5).

## Results and discussion

To evaluate MUMAL2, the parameters were kept with default values, i.e., number of nodes in the hidden layer = 4, momentum = 0.2, learning rate = 0.3, and epochs = 1000. The experiments were performed on a Linux machine equipped with Intel^®^ Celeron^®^ CPU N2830 2.16 GHz × 2, and 4 GB of RAM. Our intention was to prove that MUMAL2 can provide a quick answer even on personal computers. In fact, one iteration of MUMAL2, i.e., one execution of the strategy cost matrix + ANN, takes 20 s on average. Because eleven executions to produce the final model are needed, the total time taken is 220 s, in general. We realized that it is not significantly different from MUMAL’s running time because MUMAL has also to execute a number of iterations to produce its best results. Therefore, we concentrate the analyses of our experiments on the capacity of our approach to assess PSMs.

### Measuring the predictive power of MUMAL2

**Fig. 5:**
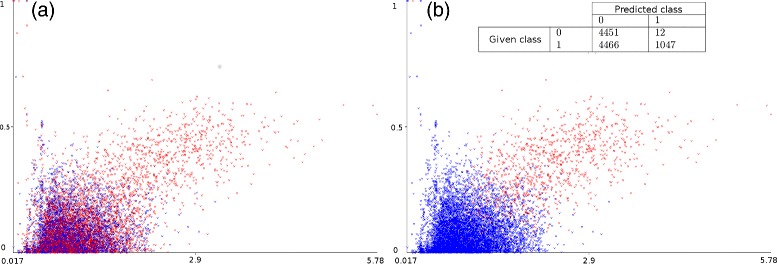
Instance relabeling provided by MUMAL2. A plot of △Cn vs Xcorr for dataset M123 is shown in **a** and **b**, before and after applying MUMAL2, respectively. Class-0 instances are shown in *blue*, while class-1 instances are shown in *red*. Part **b** includes also the confusion matrix on the top to show the huge number of class-1 instances that were classified as class 0

First, dataset M123, whose proteins are known, was used to measure the predictive power of our method. We analyzed whether the relabeling of wrong PSMs in class-1 to class-0, i.e., the establishment of a suitable decision boundary between correct and incorrect hits, could be satisfactorily accomplished. Figure 5 contains plots of △Cn vs Xcorr for dataset M123 before (Fig. 5 a) and after (Fig. 5 b) running MUMAL2. Class-0 PSMs are represented in blue, whereas class-1 PSMs are shown in red. A dense cloud of points in Fig. 5 a can bee seen composed of approximately 50% of decoys and 50% of targets. According to the target/decoy principle, this dense cloud represents the set of wrong PSMs. Therefore, we are interested in the part that is comprised mostly of red points (likely correct targets). As already mentioned, the use of a cost matrix to construct an accurate model for the class-0 instances is an attempt to keep these instances as such, since decoy PSMs are obviously wrong, whereas correctly relabeling the wrong class-1 instances, the ones mixed with decoys in the dense cloud, to class 0. Figure 5 b shows that the decision boundary produced by MUMAL2 seems to provide a good separation of the mixture of decoys/targets from the homogeneous part composed of red points. Notice that the confusion matrix on the top makes evident the huge amount of target PSMs that were classified as class 0, which was expected because most of these PSMs are known to be wrong. The plot built as a result of the instance relabeling shows that the vast majority of instances in the dense cloud turned into blue.

Even though the plot in Fig. 5 b indicates that the region of interest (the part in Fig. 5 a composed mostly of red points) could be identified, it is possible to provide more precise measurements of the quality of our solution because the proteins of dataset M123 are known. The confusion matrix on the top of Fig. 5 b shows that 12 decoy hits were mistakenly classified as class 1. Probably, some correct target hits were incorrectly relabeled to class 0 as well. Thus, to provide a more precise assessment of our strategy, the 8917 instances predicted as class 0 and the 1059 predicted as class 1 had their peptide sequences inspected to check whether or not they came from the set of expected proteins. As a result, we could build a more useful confusion matrix, considering positive or negative those instances whose peptide sequence came from the list of known proteins or from a random protein, respectively. As a consequence, we could use classical metrics that express the predictive power of an ML model, as shown in Tables 2 and 3. It can be seen that MUMAL2’s classification was highly accurate. Only 25 instances (12 negatives and 13 positives) were misclassified, also leading to very high values of sensitivity, specificity, and precision. However, it is important to highlight that the decoy hits are known to be wrong PSMs. Therefore, the 12 misclassified decoys shown in Fig. 5 b have no importance, i.e., only target instances are further considered after the classification.

**Table 2:** Assessing MUMAL2 according to the known proteins of datset M123

		Predicted class	
		0	1
Actual class	0	8904	12
	1	13	1047

A confusion matrix is shown, where positive and negative instances are not target and decoys anymore. Instead, an instance is considered positive if its peptide sequence came from the list of known proteins. Otherwise, the instance is considered negative

**Table 3:** Assessing MUMAL2 according to the known proteins of datset M123

Statistical measures	
Accuracy	0.9975
Sensitivity	0.9877
Specificity	0.9987
Precision	0.9887

It is clear, thus, that the application of a supervised ML method here does not follow the classic steps: Build the learning model using a training set, and apply the resultant model to unknown instances. In our case, the ANN is trained and applied using the same data. Notice that the target instances are the ones of interest. Our final aim is to separate wrong targets from correct targets. To this end, we use a higher cost for FPs to force the model to learn how to correctly classify decoys whose labels are obviously correct. That is why we say that decoys help to pin down wrong targets. Next, we apply the final model on the same data to relabel the wrong target hits, i.e., the ones with similar features to decoy hits, to class 0. Thus, it does not make sense to talk about cross-validation to evaluate the model. Instead, we have to verify whether correct and incorrect targets are being identified.

**Fig. 6:**
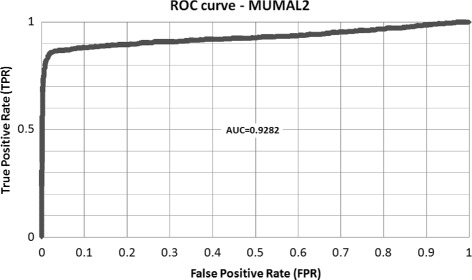
ROC curve of MUMAL2’s model for dataset M123. An instance is regarded as TP if its peptide sequence came from the list of expected proteins. Otherwise, the instance is considered FP

Another important aspect to analyze is whether the probabilities produced by our model are coherent. This is a very relevant matter if the intention is to use a method for protein inference that takes PSM probabilities into account. As shown in Fig. 3, the AUC and other measures are low because the wrong target hits that are correctly relabeled to class 0 are counted as misclassification. In fact, the blind application of MUMAL2 to dataset M123 leads to an AUC of 0.60. However, as we know the proteins of dataset M123, we can produce the real ROC curve to evaluate the probabilities generated by TSA. Figure 6 shows the ROC curve built for dataset M123 counting as TPs those instances whose peptides came from the list of known proteins, and counting as FPs, otherwise. As can be seen, the AUC is very satisfactory (nearly 0.93), showing that the probability values are appropriate, and corroborates the high predictive power of MUMAL2.

**Fig. 7:**
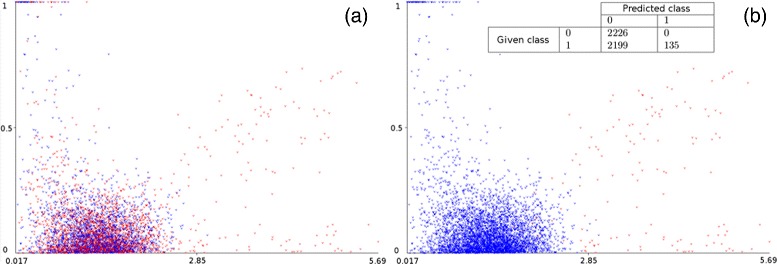
Instance relabeling performed by MUMAL2 for dataset S1_PH_CH2. A plot of △Cn vs Xcorr for the dataset is shown in **a** and **b**, before and after using MUMAL2, respectively. Class-0 hits are shown in *blue*, whereas class-1 hits are shown in *red*. Part **b** addtionally presents the confusion matrix on the top, demonstrating the significant number of class-1 examples that were classified as class 0

MUMAL2 was also applied to the other datasets for which the proteins are not known a priori. However, it is possible to use plots of △Cn vs Xcorr, as previously shown for dataset M123, to perform a visual inspection. Figure 7 shows this analysis for dataset S1_PH_CH2. It can be seen in the confusion matrix (Fig. 7 b) that a significant number of class-1 instances were relabeled to class 0, as expected. In the plot of Fig. 7 b, the colors of the points indicate that the classification seem to be appropriate because the blue points correspond to those in Fig. 7 a composed of a mixture of targets and decoys, i.e., the part where targets are probably wrong, according to the target/decoy principle. Performing the same analysis for the other datasets led to very similar outcomes (not shown), i.e., MUMAL2 promoted an expressive migration of target hits to class 0, resulting in a plot where class-0 instances correspond to the mixture of class-0 and class-1 instances before the application of MUMAL2.

### Comparing MUMAL2 with previously proposed methods

The next experiments demonstrate the superior sensitivity of MUMAL2 in relation to important methods for PSM assessment: MUMAL, MUDE, PeptideProphet, and bivariate decoy/target analyses, where the thresholds of two scores, often △Cn and Xcorr, leading to a desired FDR are pursued. For comparisons with phosphodata, we included a bivariate analysis with △M and Xcorr, following Beausoleil et al. and Jiang et al. recommendation [21, 33]. According to them, △Cn scores are often suppressed when phosphopeptides have more than one potential phosphorylation site. Therefore, △M should be used instead. For a detailed description of how the methods used in the comparison were run to produce the results shown next, refer to the works of Cerqueira et al. [3, 34].

**Fig. 8:**
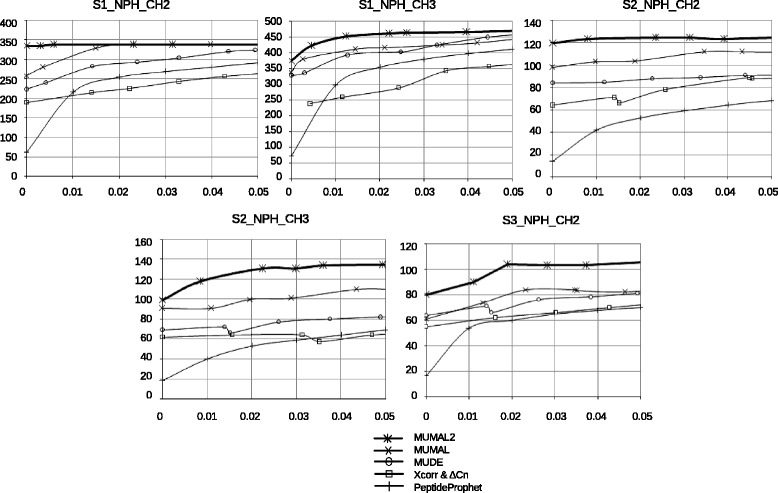
Comparing the sensitivity of MUMAL2 and other approaches with data of non-phosphorylated proteins. For each dataset, a curve of the number of identified PSMs vs estimated FDR is shown. FDR values vary from 0 and 5%

**Fig. 9:**
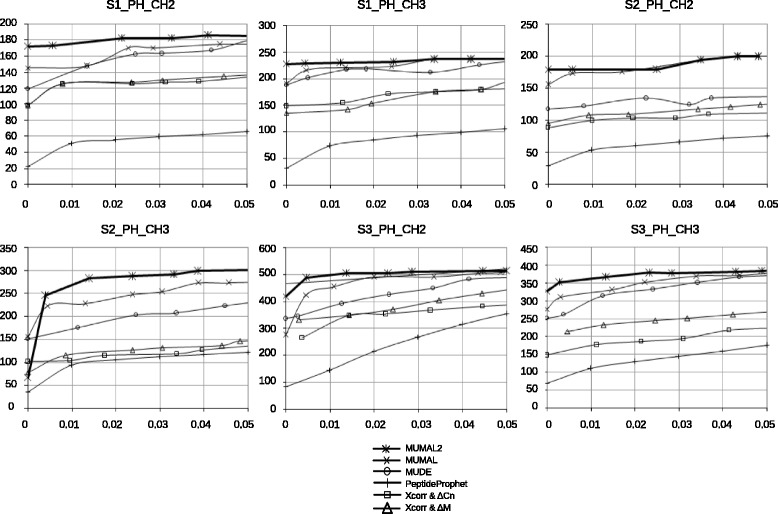
Comparing the sensitivity of MUMAL2 and other approaches with data of phosphorylated proteins. For each dataset, a curve of the number of identified PSMs vs estimated FDR is shown. FDR values vary from 0 and 5%

Figures 8 and 9 show the curves of the number of identified PSMs vs estimated FDR for all above-mentioned methods applied to the eleven datasets of unknown proteins. It is possible to build such curves because all those approaches provide an effective way to estimate the FDR value for a given set of selected PSMs. The curves show FDR values varying from 0 to 5%, which are the error rates commonly accepted. It can be seen in all cases that MUMAL2 is superior than MUDE, PeptideProphet, and the bivariate analyses.

Regarding MUMAL, MUMAL2 has an equal or greater sensitivity. It is expected because MUMAL2 performs 10 executions with CFP varying from 1 to 10. The execution with CFP = 1 is equivalent to the MUMAL execution. Therefore, MUMAL2 cannot be worst, but it can eventually present the same sensitivity as MUMAL. Notice, however, that the number of cases where MUMAL2 has a greater sensitivity is higher than the cases of equal performance. Our method could provide an average increase of 16.5 and 7.2% in relation to MUMAL for non-phosphodata and phosphodata, respectively. It means about 24 and 20 more peptides, on average, respectively.

In particular for a 1% FDR, which is a commonly pursued FDR value, MUMAL2 demonstrates superiority in all cases. For non-phosphorylated proteins, the PSM evaluation provided by MUMAL2 for this FDR led to an average improvement in sensitivity of 16.8% compared with MUMAL, meaning about 23 additional peptides. For phosphorylated proteins, in turn, the increase was approximately 12%, resulting in nearly 31 more peptides.

**Fig. 10:**
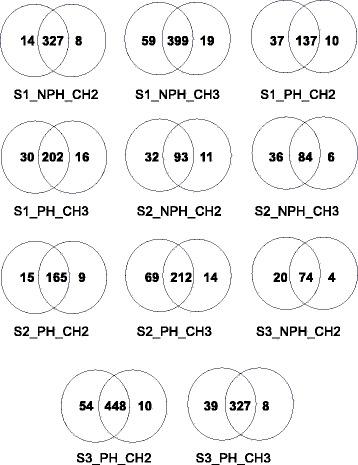
Comparison of MUMAL2 with MUMAL by Venn diagrams containing the number of PSMs selected for FDR=1%. For each diagram, the left-hand side contains the number of peptides identified exclusively by our method, while the right-hand side shows the number of identifications found exclusively by MUMAL

Figures 8 and 9 demonstrate that MUMAL presented the best performance among the methods being compared with our approach. For this reason, we performed an additional experiment to compare MUMAL2 with MUMAL by means of Venn diagrams to call attention to the higher number of exclusive peptide identifications provided by the former. Figure 10 shows this counting for a 1% FDR in both methods. In all cases, an expressive superiority of MUMAL2 can be noted. On average, the number of exclusive PSMs that our method could find is almost 4-fold greater. This is an important result because more peptides may imply more identified proteins and a higher proteome coverage.

## Conclusions

The target-decoy database strategy is widely used for data analysis in shotgun proteomics. Many previous studies have demonstrated the effective capacity of this approach for FDR estimation. However, the classical TDDB procedure does not take sensitivity into account. Fortunately, this fact has been changing since the introduction of MUDE and MUMAL.

In this work, we have further improved sensitivity in MS/MS-based peptide/protein identification by using advanced machine learning methods that use decoys to establish more appropriate decision boundaries. Furthermore, the probabilities assigned to PSMs by our method are proven to be highly accurate. This is a fundamental matter to improve protein inference when the applied approach depends on such probability values, as in the case of ProteinProphet.

We could demonstrate that our new approach has great potential to provide important improvements in protein identification, which will impact future studies that seek a broader understanding of notable cell activities. Hopefully, future research on drug discovery, diseases, and many other studies in life sciences will be positively affected by this new computational strategy for peptide/protein identification.

## Abbreviations

MS/MS: Tandem mass spectrometry
LC-MS/MS: Liquid chromatography coupled with tandem mass spectrometry
PSM: Peptide-spectrum match
DB: Database
FDR: False discovery rate
TDDB: Target-decoy database
ANN, Artificial neural network; ROC: Receiver operating characteristic
AUC: Area under the curve
TSA: Threshold selector algorithm
RT: Retention time
CFP: Cost of a false positive
CFN: Cost of a false negative
